# The Asymmetrical Effects of Divided Attention on Encoding and Retrieval Processes: A Different View Based on an Interference with the Episodic Register

**DOI:** 10.1371/journal.pone.0074447

**Published:** 2013-09-09

**Authors:** Jonathan Guez, Moshe Naveh-Benjamin

**Affiliations:** 1 Department of Psychology, Achva Academic College, Arugot, Israel; 2 Beer-Sheva Mental Health Center, Beer Sheva, Israel; 3 Department of Psychological Sciences, University of Missouri, Columbia, Missouri, United States of America; University of Melbourne, Australia

## Abstract

In this study, we evaluate the conceptualization of encoding and retrieval processes established in previous studies that used a divided attention (DA) paradigm. These studies indicated that there were considerable detrimental effects of DA at encoding on later memory performance, but only minimal effects, if any, on divided attention at retrieval. We suggest that this asymmetry in the effects of DA on memory can be due, at least partially, to a confound between the memory phase (encoding and retrieval) and the memory requirements of the task (memory “for” encoded information versus memory “at” test). To control for this confound, we tested memory for encoded information and for retrieved information by introducing a second test that assessed memory for the retrieved information from the first test. We report the results of four experiments that use measures of memory performance, retrieval latency, and performance on the concurrent task, all of which consistently show that DA at retrieval strongly disrupts later memory for the retrieved episode, similarly to the effects of DA at encoding. We suggest that these symmetrical disruptive effects of DA at encoding and retrieval on later retrieval reflect a disruption of an episodic buffer (EB) or episodic register component (ER), rather than a failure of encoding or retrieval operations per se.

## Introduction

Much research over the past decade has investigated the similarities and the differences between encoding and retrieval. There are well-known assumptions and theories regarding the idea that effective retrieval must reflect the specific manner in which the event was originally encoded. This notion clearly underlines the concepts of the encoding specificity principle [1], which states that items are encoded in a highly specific way, and that effective retrieval cues must reflect this specificity. The notion of a necessary overlap between encoding and retrieval is also found within the proceduralist approach [2], and the transfer-appropriate processing approach [3].

However, there are also established findings that highlight the asymmetry between encoding and retrieval by showing that episodic memory is easily disrupted when attention is divided during encoding, but less so, if at all, during retrieval [4], [5], [6], [7], [8]. Using varied manipulations (for example, resource allocation instructions) and secondary task costs, the overall conclusion has been that encoding is a more controlled process that competes with secondary task demands, and therefore causes memory performance decreases. By contrast, retrieval can be seen as a more obligatory process that runs relatively uninterrupted while drawing on significant attentional resources. Later, several studies have shown that under conditions where the secondary task competes at the representational level (structural interference) with the primary task (e.g. both require verbal codes), retrieval is also affected by the concurrent task [9], [10], [11]. However, the basic asymmetry still remains (the effect of DA at retrieval is not as severe as that associated with DA at encoding; e.g. [12]).

The studies above refer to the term “encoding” as a synonym for “study” or “learning” and to “retrieval” as “test”. The basic methodology uses serial stimuli presentation (in a variety of presentation modes) and after some interpolated task to avoid recency effect, a test phase is conducted (ranging from recognition to recall tasks). DA is then attached to learning (DAF – Divided Attention/Full attention), to test (FDA – Full attention/Divided Attention), compare to full attention condition (FF – full attention at learning and full attention at test). This association between encoding and learning and between retrieval and test is widely used in the literature and appears in cognitive [4]–[11] as well as neuroimaging studies (e.g. [13], [14]). However, it is also widely agreed upon that any experience (tasks) carries encoding consequences, and that these consequences will depend on the type, depth, attention, etc. of the actual operations. In that sense, retrieval-test tasks include episodic encoding or episodic registration. This confound in the terminology leads us to propose an alternative view, in which episodic consequences should be mediated by an episodic buffer (EB) or episodic register (ER) playing a role in Baddelley’s working memory (WM) model rather than ‘encoding’.

In order to strengthen the claim that it is not ‘encoding’ per se that is affected under DA conditions, research fails to define the exact mechanism disrupted under DA. The finding suggests that a decrease in levels of processing [15], [16], in self-generation [15], in associative processing [17], or in processing time (as found by the calibration functions in [5], [18]), cannot fully explain the decrease in memory performance under DA at encoding. In the current study we suggest that an on-going process, such as the Episodic register (ER), is the missing part in the encoding/retrieval asymmetry debate. Baddeley [19] proposed an episodic component in working memory subserving as the linkage between WM and long term memory (LTM). The episodic buffer is assumed to be a limited capacity storage serve as manipulating information registered system, which makes it a good candidate for DA vulnerability [19], [20]. It is assumed to play an important role in feeding information into and retrieving information from episodic LTM.” [19]. This notion of EB, or what we suggest as an Episodic Register (ER), is independent from the nature of the process conducted by a subject either at encoding or retrieval. Taking into consideration that DA seems to affect episodic memory but not semantic memory, it seems that a component related to episodic registration might be the locus of the detrimental effects of DA at encoding. Furthermore, we suggest that the ER component is not tied to encoding or retrieval per se but evoked at both as an ongoing process.

### Current Study Hypothesis and Design

In the current work we test the hypothesis that DA disrupts the ER component as well as consequent remembering, rather than encoding per se. As such, DA is detrimental to future episodic remembering either for encoding or retrieval experiencing. How can we then explain the robust asymmetry in the effects of DA at encoding and retrieval based on this view? The question is why previous studies that manipulated DA at encoding and retrieval have mainly shown episodic memory disruption mainly under DA at encoding? One reason could be the fact that the only way to test episodic remembering is *after* the event had occurred. In this sense, there is an inherent asymmetry in the way previous research assessed effects of DA at encoding and at retrieval. The basic paradigm employed tested memory for the encoding phase after encoding was complete and became ‘past experience’. However, testing the retrieval phase was performed ‘online’. This created an asymmetry in the paradigm used that might have resulted in the asymmetry in the effects of DA, as previous research potentially created a confound between the memory phase (encoding and retrieval) and the memory requirements (memory “for” encoded information vs. memory “at” test).

One way to control for this confound is to compare the effects of DA at encoding and at retrieval using the same memory requirements, for example by employing only memory for the information. This can be accomplished by testing the effects of DA at encoding and at retrieval after both have occurred: conducting a first test where the effects of DA during the original encoding can be assessed, as done previously, and in addition, conducting a second test where the effects of DA during retrieval in the first test, can be assessed. This methodology used in fMRI analysis [21] found that testing a participant in a second test for the distractor that appeared in the first test showed that those items that were later remembered were related to greater activity in the left frontal regions rather than those that were later forgotten. This pattern suggests involvement of ER in processing the distractors although not encoding them in the traditional use of the term.

A recent study by Dudukovic et al. [22] employed such a methodology, but it too used overlapping interpretation simultaneously referring to retrieval as a separate process and as an encoding process, thus falling in the same pitfalls as previous studies. As mentioned above, this overlapping interpretation stems from literature classifications that have been categorically labeled as either primarily tapping encoding or retrieval, with interpretation following from such binary classification. Furthermore, Dudukovic and her collogue did not rule out the possibility that participants were used to intentional learning during the test phase, and thus their effects can actually be related to the DA effect on intentional learning. They also did not compare the actual effect of DA during learning, rendering impossible the direct comparison of the DA effect during learning and test.

Another relevant extension we suggest here is related to the analysis of results. In their work, Dudukovic and her collogues separately analyzed test 1 and test 2. We believe that the appropriate analysis is to add the two tests as a factor in the model. This will allow a direct comparison and provide the opportunity to reveal possible interactions between attention and stimuli/test conditions.

In this paper we first challenge the encoding/retrieval asymmetry under divided attention conditions, pointing to a methodological confound in the literature of asymmetry. Second, in order to avoid the confound between referring to encoding as retrieval and retrieval as encoding, we refer to the episodic register as the missing component in the above mentioned researches. Accordingly we suggest that this episodic component is affected under DA.

We hypothesize that DA at retrieval will cause a decrease in the episodic remembering of the retrieval phase, similar to the decrease found in episodic remembering of the encoding phase. That is, although DA at retrieval had been previously shown to result in only small decrements, if any, in memory performance, our claim is that such DA effects at retrieval have detrimental consequences in terms of the ability to retrieve this information at a later time. If this is the case, it could indicate that both encoding and retrieval are interrupted by DA and that the asymmetry found in the literature could be due to an asymmetry in the test employed (“for” vs. “at”). If episodic remembering of the retrieval phase is not affected by DA at retrieval, this would provide further support for the previously suggested inherent asymmetry between encoding and retrieval processes.

Testing memory for a retrieved event raises a number of unique methodological challenges. First, if a second memory test is used to assess the effects of DA during the first memory test, participants may rely on their memory from the original encoding rather than on their episodic remembering of the first test events. This may mask any effects of episodically remembering of the retrieval event, since participants would be able to retrieve targets from the initial learning phase rather than from the retrieval phase. A second potential problem is that retrieving targets in the first test can enhance their later memorability in comparison to the initial encoding phase, which acts of memory retrieval encourage further episodic, as noted by many [23], [24], [25].

In order to assess the effects of DA on memory “for” the encoded and retrieved information, while addressing the above-mentioned methodological issues, we employed a recognition memory paradigm in the following series of experiments, and tested memory “for” retrieved information by using a second memory test, where participants had to retrieve the distractor items from the first retrieval phase. This controls for the methodological flaw noted above, that would have been created if targets retrieved from the original encoding were also to appear in the second test. The use of distractors only as representatives of the first retrieval phase is supported by studies showing that people use similar retrieval processes (a retrieval mode, a search component, and a decision component) for assessing targets and distractors during a recognition test. For example, Kapur et al. [26] found that the retrieval mode remains the same for target and distractors. Likewise, Naveh-Benjamin, Guez and Marom [27] showed similar attentional costs associated with the retrieval of targets and distractors.

The following four experiments employed a procedure in which lists of single words were studied under full or divided attention, either at encoding or at retrieval. A second test was subsequently employed under full attention, where some of the distractors from the first test (that appeared either under full or divided attention conditions) were presented as targets, and participants had to recognize them among new distractors. Different learning instructions (intentional vs. incidental) were employed in the different experiments and measures of memory accuracy, as well as retrieval latency, and secondary task costs, were collected.

## Experiment 1

The main goal of the first experiment was to test the above hypothesis using a design different than the one used in previous experiments. The new design involved the adding of an additional second test phase where memory for some of the materials that appeared in the first test were assessed. This leads to three experimental conditions. A DA manipulation was used at the learning phase (DA-F-F, e.g. Divided attention at learning, followed by free attention at test, and free attention in the following ‘re-test’), in the first test/retrieval (F-DA-F, e.g. free attention at learning, followed by DA at test, and free attention in the following ‘re-test’) phase or in none (F-F-F, e.g. free attention in all learning and tests phases).

### Method

#### Participants

Twenty-two undergraduate students from Ben-Gurion University of the Negev took part in the experiment for course credit. The study was approved by the local institutional review board of Ben-Gurion University. All participants gave their written informed consent for study participation.

#### Design

Two independent within-subject variables were used. The first was attention with three levels (F-F-F, DA-F-F and F-DA-F). The second retrieval test served to measure memory for the first retrieval test, thus it was always performed under full attention. A second independent variable was the stimuli to be remembered with three levels. The first included target words from the study list that were tested in the first test. The second level included target words (other than the former) from the study list that were tested in the second test. Finally, there were distractors from the first test that were served as targets in the second test (see table 1). The dependent variables were proportion of correctly recognized targets (percentage hits minus percentage FA) on the first test, and the proportion of correctly recognized targets of the second test (both for those words that served as distractors on the first test, as well as the words from the initial study list). In addition to memory accuracy, performance on the secondary task was measured (both alone as a baseline, and under DA at encoding or at retrieval during the first test).

**Table 1:** Study tests list design in the four experiments.

Experiment 1 & 3			Experiment 2 & 4		
Learning	Test 1	Test 2	Learning	Test 1	Test 2
L1	D1	D3	L1	D1	D3
L2	D2	L4	L2	D2	Dt2-1
L3	L5	D1	L3	L5	D1
L4	L3	L2	L4	L3	Dt2-2
L5	D3	L6	L5	D3	Dt2-3
L6	L1	D2	L6	L1	D2
:	:	Dt2-1	:	:	:
:	:	Dt2-2	:	:	:
:	:	Dt2-3	:	:	:
:	:	:	:	:	:
Ln	Ln/Dn	Ln/Dn/Dt2-n	Ln	Ln/Dn	Dn/Dt2-n

Ln = learning stimuli; Dn = Distractors on test 1; Dt2-n = Distractors on test 2. Note that learned stimuli that appeared on test 2 did not used at test 1.

#### Stimuli

The words used for the memory task included 260 common concrete nouns taken from Hebrew norms [28], [29]. We created three sets of lists, each including a study and two tests. For each list, 30 words were used for the study phase. For the first test phase, 15 new words were used as distractors and 15 words from the study phase were used as targets. For the second test phase, 20 new words were used as distractors, and 20 words, 10 from the study phase (those that were not used in the first test), and 10 from the first test (those used as distractors in the first test), were used as targets. Accordingly, in both tests, participants exposure to the same proportion of targets and distracters (e.g. 50%). The stimuli were presented on the computer screen for two seconds each with two seconds interval before the next stimulus appeared on the screen. The same rate of presentation was used during the tests, with each new test stimulus appearing at the end of the four seconds interval.

The secondary task was an auditory continuous reaction time task (ACRT) that involved three tones (high, medium, and low pitched) and manual responses on the computer keyboard, where participants’ response caused the next tone to appear. The goal was to carry out the task quickly and accurately as possible. Participants’ responses were recorded by computer.

#### Procedure

Each participant was presented with three lists, one for each of the three attention conditions. In addition, each participant performed the ACRT alone as a baseline task twice, each time for 60 seconds: once at the beginning and again at the end of the experiment. Between the study phase and the first test phase and between the first test phase and the second test phase, participants were engaged in 30 seconds interpolated activity in which they had to continuously subtract by multiples of seven from a random three-digit number that appeared on the screen, in order to eliminate recency effects on memory. After this interpolated task, the first recognition test phase began in which participants were instructed to say whether or not a presented stimulus had already appeared during the study phase. After another interpolated activity, the second recognition test phase began in which participants were instructed to say whether or not a presented stimulus had already been seen before in the study or the first test phases. The order of the words during the study and the test phases for each list was randomized for each participant.

Under the full attention condition, participants were told to pay full attention to the words in order to encode and retrieve them. In the ACRT baseline condition, participants were instructed to respond as fast and as accurately as they could. In the divided attention conditions, they were told to pay equal attention to encoding or retrieval and to the ACRT task. Before each list, participants were told which attention condition to expect.

There were four experimental tasks:

1. Single-task performance: memory full attention. In this task, participants were instructed to study information under full attention conditions, then perform the first recognition test under full attention and finally perform the second recognition test.
2. Single-task performance: ACRT task. Participants performed the ACRT task for 60 seconds (two trials, at the beginning and at the end of the experiment).
3. Dual-task: divided attention at encoding. In this task, participants performed the encoding and the ACRT task simultaneously, under instructions to pay equal attention to each. After the study phase, the two tests were administered as in the full attention condition.
4. Dual-task: divided attention at retrieval. In this task, participants encoded information under full attention, and then performed the first recognition test and the ACRT task simultaneously, under instructions to pay equal attention to each. The second test was then administered under full attention as in the other memory conditions.

Participants initially practiced the ACRT task alone, the memory task alone (full attention), and their combination either at encoding (DA at encoding), or at retrieval (DA at retrieval). They then continued with the experimental trials. The order of the tasks was counterbalanced across subjects.

### Results and Discussion

#### Memory performance for learned and tested items

**Figure 1:**
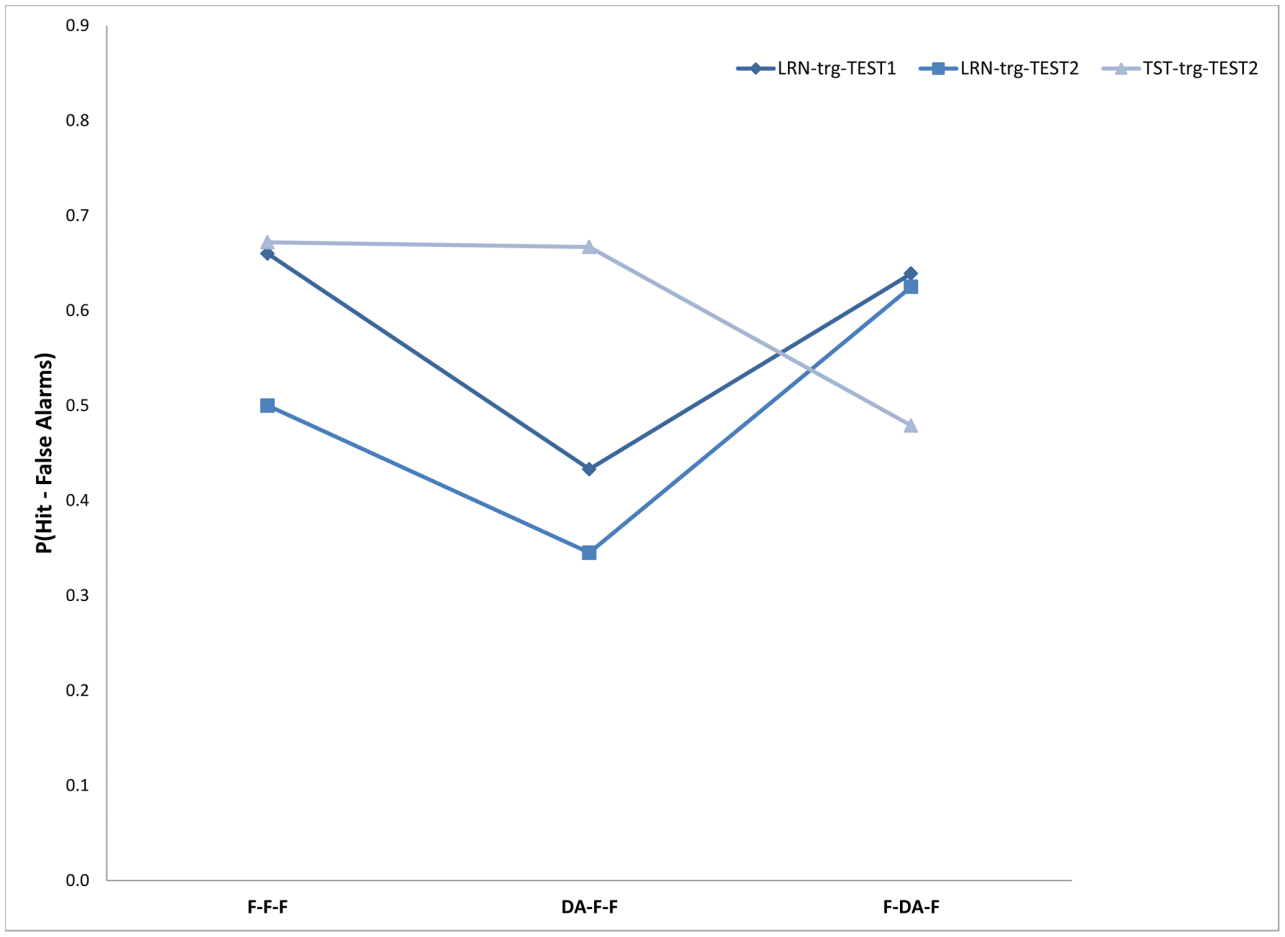
Memory performance as a function of the targets to be retrieved under the different attention conditions.

Mean percentage of words recalled correctly (percentage hits minus percentage FA) across participants for each condition appears in Table 2. A two-way ANOVA with attention conditions (three levels) and memory target conditions (three levels) showed the effects of attention and memory target to be significant, F(2,42) = 5.74; Mse = 0.05; p<0.01 and F(2,42) = 5.79; Mse = 0.04; p<0.01, respectively. The effect of the interaction was also significant, F(4,84) = 18.55; Mse = 0.02; p<0.01, see Figure 1.

**Table 2:** Means of proportion correct response (proportion Hit minus proportion FA) for the three memory measures – target types at the three attention conditions: Experiment 1.

	Attention condition					
	F-F-F		DA-F-F		F-DA-F	
Measure	*M*	*SD*	*M*	*SD*	*M*	*SD*
Memory performance for the learning phase in test 1	0.66	0.22	0.43	0.20	0.63	0.25
Memory performance for the learning phase in test 2	0.50	0.23	0.34	0.21	0.62	0.23
Memory performance for the distractors of retrieval phase in test 2	0.67	0.18	0.67	0.17	0.47	0.22

Further comparisons showed that remembering items from the encoding phase in the first test was not different from the F-F-F (Mean 0.66) and from the F-DA-F (Mean 0.64) conditions, F<1, while performance in the DA-F-F condition (Mean 0.43) was significantly lower than both, F(1,21) = 15.79; Mse = 0.036; p<0.01, and F(1,21) = 8.33; Mse = 0.056; p<0.01, respectively, for the F-F-F and F-DA-F conditions.

Similar patterns were found in remembering items from the encoding phase of the second test. Comparison of the F-F-F condition (Mean 0.50) and the F-DA-F condition (Mean 0.62) was not significant, F(1,21) = 4.12; Mse = 0.038; p>.05. However, performance in the DA-F-F condition (Mean 0.34) was significantly lower than performance in both the F-F-F and the F-DA-F conditions, F(1,21) = 13.54; Mse = 0.02; p<0.01, and F(1,21) = 43.06; Mse = 0.02; p<0.01, respectively.

An opposing pattern was found for remembering items from the first test phase (remembering the distractors from the first test). In this condition, no difference was found between the F-F-F (Mean 0.67) and DA-F-F conditions (Mean 0.68), F<1, while performance in the F-DA-F condition (Mean 0.48) was significantly lower than both F(1,21) = 17.21; Mse = 0.023; p<0.01, and F(1,21) = 12.83; Mse = 0.033; p<0.01, respectively.

Overall, memory performance for remembering items from the encoding/study phase under the different attention conditions closely replicates previous results showing that DA at encoding interferes with memory performance (relative to full attention) while DA during the retrieval/test phase show no such a decrease. By contrast, memory performance for items from the first retrieval/test phase was negatively affected only in the DA at retrieval condition relative to the other conditions. These findings suggest that retrieval is not immune to DA effects but that the impact of the DA interference effects at retrieval can be seen only in future test performance.

#### ACRT task performance

The ACRT task was performed alone (under full attention) as a baseline measure and in the DA conditions, once during the encoding/study phase and once during retrieval/test phase. The mean average RT was 772 msc. (175) for the baseline condition and 1231 (268), and 1532 (469), for DA at encoding and retrieval, respectively.

A one-way ANOVA analysis yielded a significant attention effect [F(2,42) = 73.35, MSe = 43915, p<0.01]. Post hoc comparisons showed that all the pair comparisons were statistically significant. RT in the baseline condition was significantly lower than that in the DA at encoding, and the DA at retrieval [F(1,21) = 112.3, MSe = 20587, p<0.01 and F(1,21) = 90.96, MSe = 69835, p<0.01 respectively]. Average RT for DA at encoding was significantly lower than DA at retrieval [F(1,21) = 24.18, MSe = 41323, p<0.01].

These results replicate previous findings, indicating that both encoding and retrieval are attention demanding processes (requiring substantial attentional costs relative to the baseline condition), and that retrieval is especially attention demanding [5].

The importance of the results of Experiment 1 is in showing that when a different testing design is used (in which testing for the effects of DA at retrieval is done later on, as at encoding), DA at retrieval does seem to significantly impair memory performance. In this sense, these data strongly indicate the conclusions reached in previous research, namely, that retrieval processes are immune to DA interference, are inadequate.

In the second experiment, we wanted to replicate Experiment 1′s results while employing some methodological modifications. One potential criticism of the results of Experiment 1 is that the four seconds allotted for retrieval of each item on the first test could have been used by the participants to encode and elaborate on the stimuli, which in turn would have affected their performance on the second test. If this had been the case, then the decline in performance in the second test as a function of DA in the first could be due to a failure to encode the test stimuli in the first test. For example, participants could have responded in the first test after one or two seconds, and then spent the rest of the time to encode the test stimuli by elaborating on it. To control for this possibility, the procedure was designed such that as soon as the participant provided a response in the first test, new test stimuli were immediately presented to her/him, thereby reducing the participant’s ability to encode/elaborate on the stimuli during the retrieval phase of the first test.

## Experiment 2

### Method

#### Participants

Sixteen undergraduate students from Ben-Gurion University of the Negev took part in the experiment for course credit. The study was approved by the local institutional review board of Ben-Gurion University. All participants gave their written informed consent for study participation.

#### Design

The design was identical to the one used in Experiment 1 except that we used only two levels for target words. One level included words from the study list that were tested in the first test, and the other level included words (distractors) from the first test that were tested on the second test. The third level of target words from Experiment 1, which included target words from the study list that were tested in the second test, was eliminated. In addition, we added in this experiment a measure of response latency.

#### Stimuli

The words used in the memory task were 420 common concrete nouns taken from Hebrew norms as in Experiment 1. We created six lists, each including a study and two tests phases. For each list, 30 words were used for the study phase. For the first test phase, 20 new words were used as distractors and 20 words from the study phase were used as targets. For the second test phase, 20 new words were used as distractors, and the 20 words used as distractors in the first test, were used as targets.

At study, the stimuli were presented on the computer screen for two seconds and after a two second delay, the next stimulus appeared on the screen. For these tests, stimuli were presented for two seconds followed by a dark screen that was replaced by the next stimuli as soon as the participant responded. The secondary task was the auditory continuous reaction time task (ACRT) used in Experiment 1.

#### Procedure

The procedure was the same as the one used in Experiment 1.

### Results and Discussion

#### Memory performance for learned and tested items

**Figure 2:**
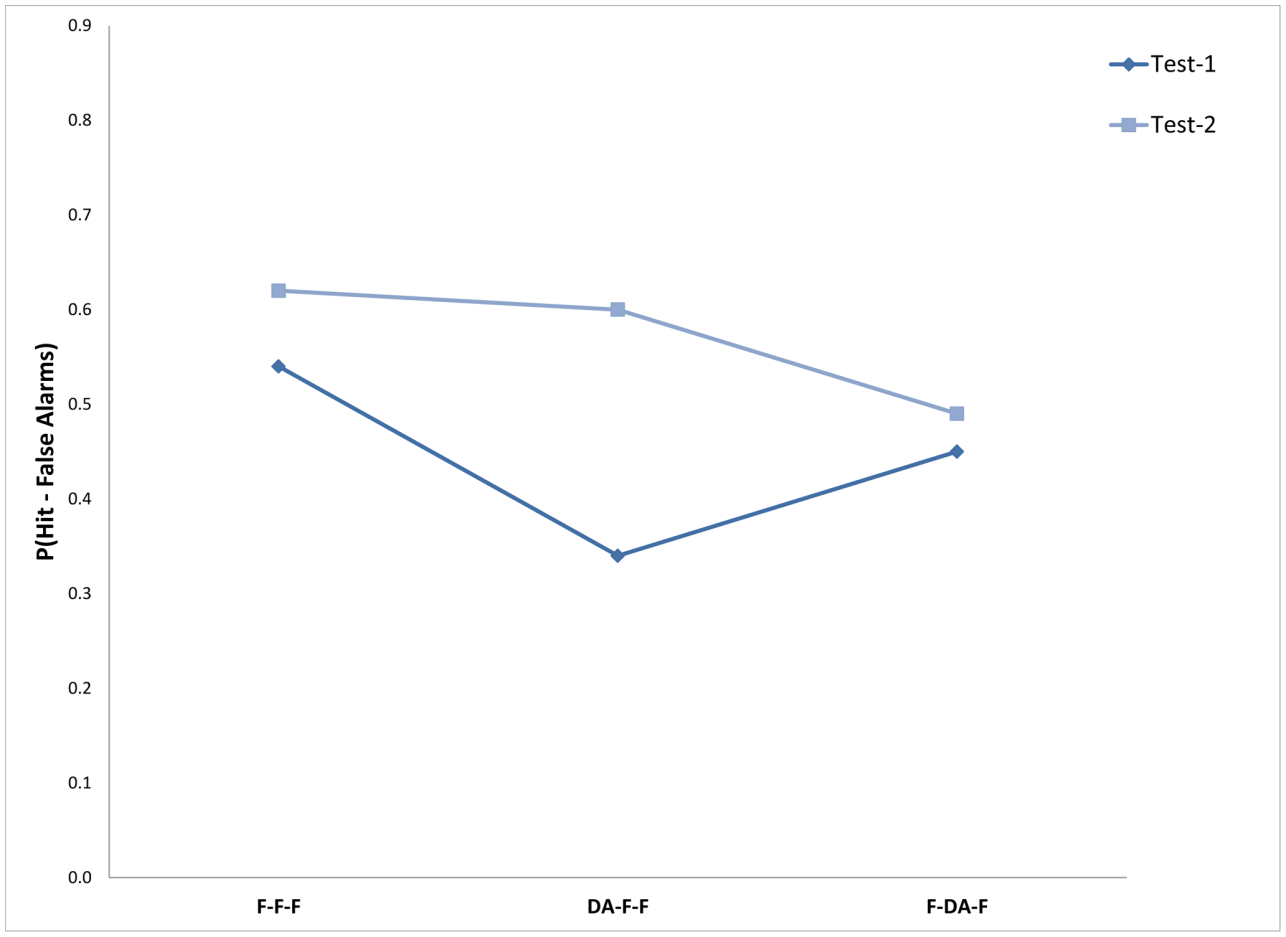
Memory performance as a function of the targets to be retrieved under the different attention conditions: Experiment 2.

Means for proportion correct response (proportion hit minus proportion FA) was computed across participants for each condition (see Table 3). Two-way ANOVA with attention conditions (three levels) and memory target conditions (two levels) showed the effects of attention and memory target to be significant, F(1,30) = 10.18; Mse = 121.7; p<0.01 and F(2,30) = 15.12; Mse = 277; p<0.01, respectively. The effect of the interaction was also significant, F(2,30) = 17.37; Mse = 65.7; p<0.01, see Figure 2.

**Table 3:** Means of proportion correct response (proportion Hit minus proportion FA) for memory measures – target types at the three attention conditions; and mean latencies (in msc.) across trials for each condition: Experiment 2.

	Attention condition					
	F-F-F		DA-F-F		F-DA-F	
Measure	*M*	*SD*	*M*	*SD*	*M*	*SD*
Memory performance for the learning phase in test 1	0.54	0.16	0.34	0.12	0.45	0.19
Memory performance for the distractors of retrieval phase in test 2	0.62	0.23	0.61	0.16	0.49	0.20
Latency at test 1	1201.4	90.3	1301.3	161.4	1756.0	288.8
Latency at test 2	1148.3	131.6	1166.1	116.7	1187.7	126.5

Further comparisons showed that remembering items from the encoding phase in the first test was significantly higher in the F-F-F condition (Mean 54.2) than in the DA-F-F condition (Mean 34.1), F(1,15) = 41.17; Mse = 78.9; p<0.01, replicating previous results showing a large detrimental effects of DA at encoding. In addition, in this experiment performance of the F-DA-F condition (Mean 45.1) was also lower than in the F-F-F condition, F(1,15) = 7.33; Mse = 89.5; p<0.05. Finally, performance in the DA-F-F condition was significantly lower than in the F-DA-F one, F(1,15) = 17.00; Mse = 57.9; p<0.01, replicating previous results of the larger decline in memory performance under DA at encoding than DA at retrieval.

An opposite pattern was found for remembering items on the second test (remembering the distractors from the first test). In this test, no difference was found between the F-F-F condition (Mean 62.3) and the DA-F-F condition (Mean 60.9) F<1, while performance on the F-DA-F condition (Mean 49.8) was significantly lower than both, F(1,15) = 14.85; Mse = 84.1; p<0.01, and F(1,15) = 8.78; Mse = 112.0; p<0.01, respectively.

These results indicate that memory performance for remembering items from the encoding/study phase under the different attention conditions closely replicates previous results showing that DA at encoding interferes with memory performance while DA during the retrieval/test phase show less such a decrease in memory performance in comparison to the full attention condition [5].

On the other hand, memory performance for items from the retrieval/test phase declines only in the DA at retrieval condition but not in the other attention conditions.

These findings suggest that retrieval is immune to the effects of DA only at the test time, but it is not immune to DA effects on future test performance where the interference effects can be seen.

#### ACRT task performance

The ACRT task was performed alone (under full attention) as a baseline measure and in the divided attention conditions, once during encoding/study and once during retrieval/test phase. The mean average RT was 640 msc. (122) for the baseline condition and 836 (180), and 1287 (308), for DA at encoding and retrieval conditions, respectively.

One-way ANOVA yielded significant attention effect [F(2,30) = 94.77, MSe = 18634, p<0.01]. Post hoc comparisons showed that all pair comparisons were significant. Baseline condition performance was significantly lower than that in the DA at encoding and DA at retrieval conditions, [F(1,15) = 71.00, MSe = 4331.3, p<0.01 and F(1,15) = 108.75, MSe = 30873, p<0.01 respectively]. Also average RT for the DA at encoding condition was significantly lower than that in the DA at retrieval condition, [F(1,15) = 78.89, MSe = 20697, p<0.01].

These results replicate previous findings, showing that encoding and retrieval are attention demanding processes (in comparison to the baseline condition), and indicating that retrieval is more demanding than encoding.

#### Retrieval latency measure

For each condition, we averaged the latency of all retrieval responses in each trial. Mean latencies across trials and participants for each condition appear in Table 3. A two-way ANOVA showed a significant effects of attention [F(2,30) = 59.52, Mse = 13294, p<0.01], and test [F(1,15) = 64.20, Mse = 23774, p<0.01], as well as a significant interaction [F(2,30) = 36.39, Mse = 16847, p<0.01]. Further comparisons of the interaction showed that retrieval latency in the first test clearly increases from the F-F-F, to the DFF, and to the FDA attention condition (see Table 3). For the first test, retrieval at the F-F-F was faster than at the DFF [F(1,15) = 18.36, Mse = 4349, p<0.01], and at the FDF condition [F(1,15) = 64.31, Mse = 38263, p<0.01]. Response latency was slower at the FDF than at the DFF condition [F(1,15) = 44.03, Mse = 37563, p<0.01]. By contrast, no significant differences were found in retrieval latency in the three attention condition of the second test, [F(2,30) = 1.81, Mse = 3416, ns.]^.^

The retrieval latency in the first test extends previous results in a cued-recall tasks to the recognition task used here showing that divided attention at encoding causes an increase in retrieval latency, and that divided attention during retrieval causes even a larger increase in response latency [30]. The finding of no differences in retrieval latency in the different attention condition in the second test makes sense since no divided attention manipulation was used for this test.

In sum, Experiment 2 replicates the basic findings of Experiment 1, under conditions where retrieval time was cut in half (from four to two seconds), reducing the time available for further encoding following the completion of retrieval in the first test.

Despite these methodological modifications above, it is still possible that participants try, albeit briefly, to encode the retrieved items in order to use them in the second test. For example, they may try to encode/elaborate on the information in the first test prior to providing the ‘yes/no’ recognition response, or they may encode/elaborate on the information while providing a recognition response. To rule out these possibilities we conducted experiment 3 and 4. In these experiments we employed incidental learning paradigms so as to avoid any intentional encoding during the retrieval phase of the first test. We then surprised participants with an unexpected second test.

## Experiment 3

In this experiment, we employed the basic design used in Experiment 1 with a number of important modifications. First, in order to create incidental learning conditions during the first test, we manipulated the attention factor between-subjects rather than within-subjects, as was done in Experiments 1 and 2. This allowed for the use of an unexpected second test, ruling out the possibility that participants encoded the events in the first test intentionally, as preparation for the second test. Furthermore, to increase the reliability of the results, we also changed the presentation modality of the memory task from visual to auditory, and that of the secondary task from auditory to visual.

### Method

#### Participants

54 undergraduate students from Ben-Gurion University of the Negev took part in the experiment for course credit. The study was approved by the local institutional review board of Ben-Gurion University. All participants gave their written informed consent for study participation.

#### Design

We used the same design as used in Experiment 1, except that the attention factor was manipulated between subjects, with 18 participants randomly assigned to each of the three attention conditions.

#### Stimuli

The words used in the memory task were 110 common concrete nouns taken from Hebrew norms as in the above experiments. We created one list that included a study and two tests phases. Fifty words were used for the study phase. For the first test phase, 20 new words were used as distractors and 20 words from the study phase were used as targets. For the second test phase, 30 new words were used as distractors, and 30 words, 15 from the study phase (those that were not used in the first test), and 15 from the first test (those used as distractors in the first test), were used as targets. The stimuli were presented auditorily at a pace of one every four seconds at study. At the test phases, participant responses were recorded by the experimenter who immediately pressed the relevant keyboard key (depending on the participant’s response ‘Yes’ or ‘No’). The experimenter’s response caused the next stimulus to be presented auditorily.

The secondary task was a visual continuous reaction time task (VCRT) that involved a visual display on a computer screen and a manual response on the computer keyboard. The display consisted of four boxes, arranged horizontally. An asterisk appeared at random in one of the boxes, and the participants’ task was to press the corresponding key on the keyboard. Participants’ responses cause the asterisk to move immediately to one of the other three at random. The asterisk didn’t move until participant responded. The goal of the participants was to carry out the task as quickly and accurately as possible.

#### Procedure

Each participant was presented with one list and was randomly allocated to one of the three attention conditions. The other details of the experiment were identical to those used in Experiment 1. Participants in the group allocated to the full-attention condition were told to pay full attention to the list in order to encode and retrieve the items. In the VCRT baseline condition, participants were instructed to react as fast and as accurately as possible. Participants allocated to the divided-attention conditions were told to pay equal attention to the encoding (or retrieval) of the words and to the VCRT task. Participants were also told which attention condition to expect. Participants initially practiced the VCRT task alone, the primary task alone (depending on their attention condition) and both tasks simultaneously (depending on their attention condition). During the practice phase they did not perform the second test nor were they told anything about it. After the practice phase they continued with the experimental trial which was identical to the one used in Experiment 1.

### Results and Discussion

#### Memory performance for learned and tested items

**Figure 3:**
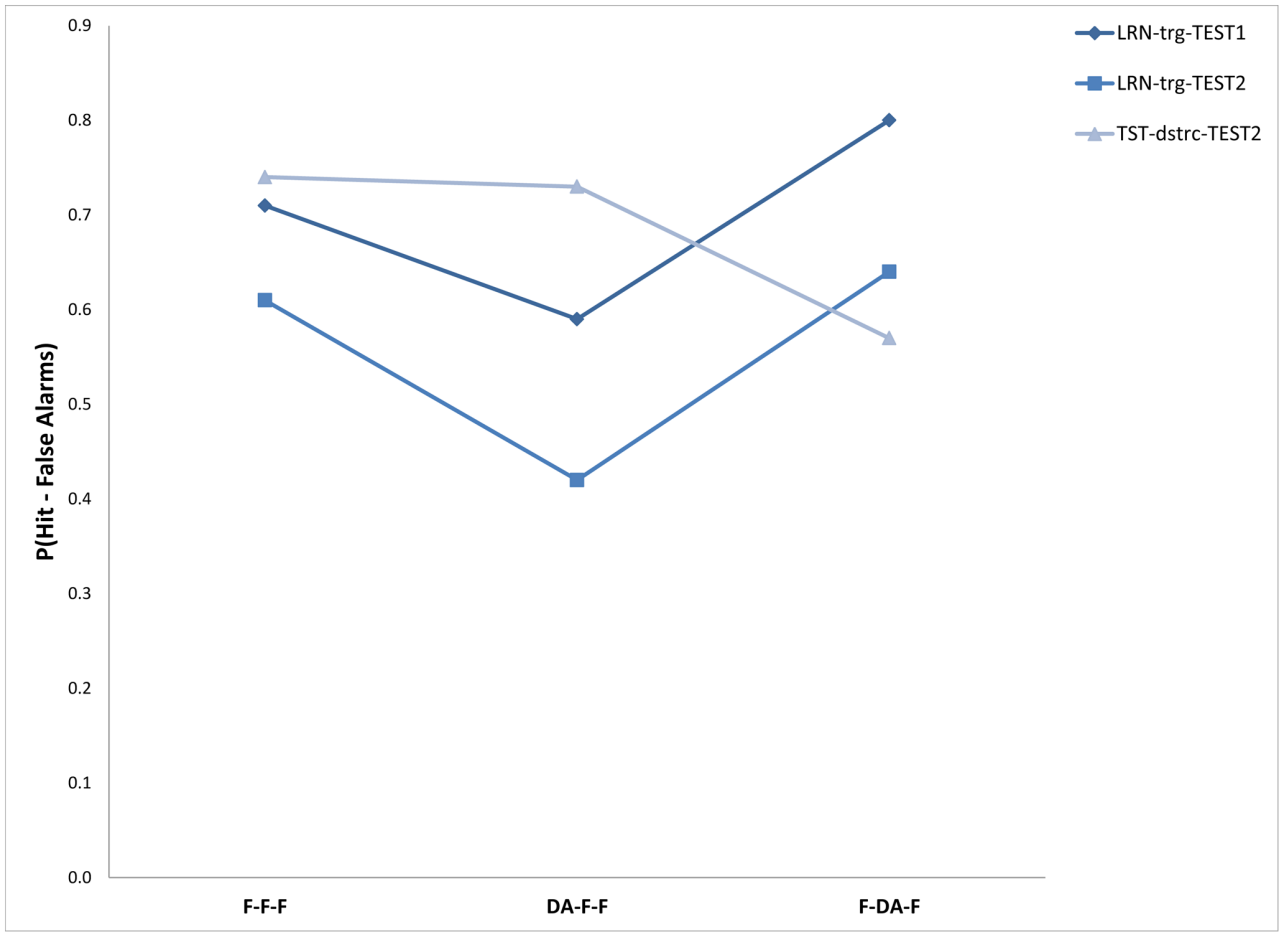
Memory performance as a function of the targets to be retrieved under the different attention conditions/groups: Experiment 3.

Mean percentage of words recalled correctly across participants for each condition appear in Table 4. A two-way ANOVA with attention conditions (3 levels) and memory target conditions (3 levels) showed that the main effect of attention was not significant, F(2,51) = 2.10; Mse = 849.4; p = 0.13, and that the main effect of the memory target was significant, F(2,102) = 14.71; Mse = 211.9; p<0.01. The effect of the interaction was also significant, F(4,102) = 11.11; Mse = 211.9; p<0.01, see Figure 3.

**Table 4:** Means of proportion correct responses (proportion Hit minus proportion FA) for the three memory measures –target types, and mean latencies (in msc.) across trials for each condition at the three attention conditions/groups: Experiment 3.

	Attention condition					
	F-F-F		DA-F-F		F-DA-F	
Measure	*M*	*SD*	*M*	*SD*	*M*	*SD*
Memory performance for the learning phase in test 1	0.71	0.16	0.59	0.15	0.80	0.12
Memory performance for the learning phase in test 2	0.62	0.17	0.42	0.20	0.65	0.28
Memory performance for the distractors of retrieval phase in test 2	0.74	0.13	0.74	0.17	0.57	0.34
Latency at test 1	1463.4	308.6	1377.8	243.8	1799.4	152.1
Latency at test 2	1510.0	213.1	1417.7	266.0	1511.7	353.7

Follow-up comparisons showed, as expected, that remembering items from the encoding phase in the first test was not different in the F-F-F condition (71.4%) and in the F-DA-F condition (80.5%) [F(1,51) = 3.60; Mse = 209.7; ns], while performance in the DA-F-F condition (59.1%) was significantly lower than in both the F-F-F and the F-DA-F conditions, F(1,51) = 6.40; Mse = 209.7; p<0.01, and F(1,51) = 19.62; Mse = 209.7; p<0.01, respectively.

A similar pattern was found for remembering items from the encoding phase in the second test (although the mean performance level for this test was lower, probably due to the longer retention interval before the second test). Performance in the F-F-F condition (61.8%) was not significantly different than that in the F-DA-F condition (64.8%), F<1, while performance in the DA-F-F condition (42.4%) was significantly lower than in both, F(1,51) = 6.76; Mse = 502.9; p<0.01, and F(1,51) = 8.98; Mse = 502.9; p<0.01, respectively.

A different (and opposing) pattern was found in the second test for remembering items from the first test phase (remembering the distractors from the first test). In this test, no differences were found between the F-F-F condition (74.4%) and the DA-F-F condition ((73.8%) F<1, while performance in the F-DA-F condition (57.0%) was significantly lower than in both, F(1,51) = 4.86; Mse = 560.4; p<0.05, and F(1,51) = 4.56; Mse = 560.4; p<0.05, respectively.

Overall, remembering items from the encoding/study phase under the different attention conditions replicated previous results showing that DA at encoding interferes with memory performance while DA during the retrieval/test phase shows no such decrease in memory performance compared with retrieval under full attention. However, in contrast, memory for items that appeared in the first retrieval/test phase was negatively affected (as measured in the performance on the second test) only in the DA at retrieval condition in the first test but not in the other attention conditions.

#### VCRT task performance

The VCRT task was performed alone (under full attention) as a baseline measure for each of the DA groups, and under the DA conditions, once during encoding/study for the DFF group and once during retrieval in the first test in the FDF group.

No differences were found between the two groups in baseline condition performance, with mean RT of 536 msec (122) for the DFF group, and 520 msec (78) for the FDF group, F<1. In addition, no difference was found between the DFF group, with a mean reaction time of 595 msec. (132), and the FDF group, 638 msec. (149), F<1. Yet, both the DA conditions resulted in higher RTs than the baseline conditions [F(1,34) = 4.74; Mse = 6751.9; p<0.05 and F(1,34) = 18.57; Mse = 6752; p<0.01 respectively]. Overall, in this experiment, encoding and retrieval exhibited demand for attentional resources to the same degree.

#### Retrieval latency

For each group, we averaged the latency of all retrieval responses in each trial. Mean latencies across trial and participants for each condition appear in Table 4. A two-way ANOVA showed a significant effect of attention/group [F(2,51) = 5.51, Mse = 112045, p<0.01], and of test [F(1,51) = 4.38, Mse = 27685, p<0.05], as well as a significant interaction [F(2,51) = 11.87, Mse = 27685, p<0.01]. Follow-up comparisons on the interaction showed no differences in response latency between the three groups on the second test (F<1). Paired comparisons between the first and second tests showed no differences for the F-F-F and the DFF group, both F<1. Yet, for the FDF group, the latency in the first test was significantly higher than in the second test [F(1,51) = 26.9, Mse = 27685, p<0.01].

The retrieval latency in the first test replicates previous finding showing that divided attention at retrieval causes an increase in retrieval latency, as reported by others [30], [31]. For the second test, we did not find this pattern, indicating that DA at retrieval does not increase latency of response on the following test for this information.

In sum, the results of Experiment 3 replicate previous findings regarding the effects of DA on encoding and retrieval; memory performance, attentional costs, and retrieval latency performance. Importantly, the results of the current experiment replicate those obtained in Experiments 1 and 2, showing that DA at retrieval does not cause a memory deficit for the retrieval of information presented at the retrieval phase, but that it does negatively affect memory of information presented during retrieval when that memory is assessed at a later test. This result was obtained even when participants did not expect a second test on the information presented in the first test. This minimizes the possibility that the results of Experiments 1 and 2 were due to participants’ expectation for an upcoming second memory test, which may have consequently caused them to try and elaborate on the information presented in the first test.

In the following experiment, experiment 4, we employed another constructive replication of the first three experiments. In Experiment 3 we used only a partial incidental learning procedure, where participants could not have suspected the upcoming second test; nonetheless, they could have learned the information intentionally in the study phase in expectation of the first memory test. In the fourth experiment, we utilized a pure incidental learning throughout the experiment. We did so by avoiding mentioning to participants that this is a memory experiment, thereby creating incidental learning conditions already at the study phase.

## Experiment 4

In order to create pure incidental learning conditions, we used a dummy instrument with lights and wires, with the wires connected to participants’ hands and elbows. The participants were told that this experiment was intended to assess their physiological reactions to presented information and that the instrument would record their galvanic skin changes and their temperature as they read the presented words. Using such a procedure assured the incidental learning of the material during the study phase, and in addition, equated the expectations of participants for the two tests, as they did not expect either of them. This is in contrast to Experiment 3, where the participants expected the first but not the second test. Furthermore, to ensure that participants were not using encoding strategies in the first test, and that the presentation time for each item was the same during the study and the first test, each word was presented for two seconds during both the study and the first test. Finally, in this experiment, we used a visual presentation for the memory task and an auditory presentation for the secondary task (ACRT).

### Method

#### Participants

45 undergraduate students from Ben-Gurion University of the Negev took part in the experiment for course credit. The study was approved by the local institutional review board of Ben-Gurion University. All participants gave their written informed consent for study participation.

#### Design

Was the same as the one used for Experiment 2, except that the attention factor was manipulated between subjects, with 15 participants randomly assigned to each of the three attention conditions.

#### Stimuli

The words used in the memory task were 100 common concrete nouns taken from Hebrew norms as in the above experiments. We created one list that included a study and two tests phases. Thirty words were used for the study phase. For the first test phase, 20 new words were used as distractors and 20 words from the study phase were used as targets. For the second test phase, 20 new words were used as distractors, and 20 words, used as distractors in the first test, were used as targets.

At study, stimuli were presented on the computer screen for two seconds each. At test, each word was presented for two seconds, after which the screen went dark until a response was made by the participant, which triggered the appearance of the next test item. The secondary task was the auditory continuous reaction time task (ACRT) used in Experiments 1 and 2.

#### Procedure

This was the same as the one used in Experiment 2 except that we used pure incidental learning instructions. Participants were connected to a dummy instrument and were told that we were interested in their physiological reactions to the presentation of words. Participants were connected to the instrument throughout the experimental session.

### Results and Discussion

#### Memory performance for learned and tested items

**Figure 4:**
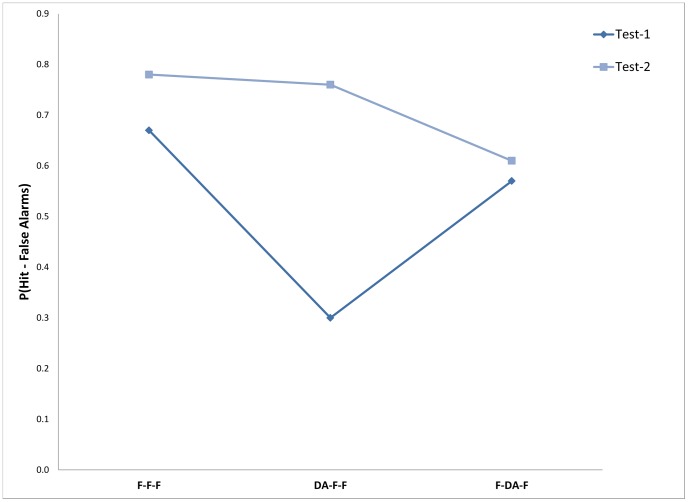
Memory performance as a function of the targets to be retrieved under the different attention conditions: Experiment 4.

Mean percentage of words recalled correctly across participants for each condition appears in Table 5. A two-way ANOVA with attention conditions (three levels) and memory target conditions (two levels) showed the effects of attention and memory target to be significant, F(2,42) = 7.36; Mse = 397.2 p<0.01, and F(1,42) = 58.4; Mse = 161.0; p<0.01 respectively. The effect of the interaction was also significant, F(2,42) = 22.68; Mse = 161.0; p<0.01, see Figure 4.

**Table 5:** Means of proportion correct responses (proportion Hit minus proportion FA) for the memory measures – target types at the three attention conditions: Experiment 4.

	Attention condition/group					
	F-F-F		DA-F-F		F-DA-F	
Measure	*M*	*SD*	*M*	*SD*	*M*	*SD*
Memory performance for the learning phase in test 1	0.67	0.18	0.31	0.14	0.57	0.20
Memory performance for the distractors of retrieval phase in test 2	0.78	0.11	0.76	0.12	0.62	0.21
Latency at test 1	1348	148	1416	147	2342	472
Latency at test 2	1255	129	1175	162	1332	169

Follow-up comparisons showed that remembering items from the encoding phase in the first test was not significantly different for the F-F-F condition (67%) and the F-DA-F condition (57%), F(1,42) = 2.60; Mse = 307.3; ns. Also, performance in the DA-F-F condition (30%) was significantly lower from both the above-mentioned conditions, F(1,42) = 32.61; Mse = 307.3; p<0.01, and F(1,42) = 16.78; Mse = 307.3; p<0.01, respectively.

A reversed pattern was found for remembering items from the first test phase in the second test phase. For this test, there were no differences in memory performance in the F-F-F (78%) and DA-F-F condition (76%) F<1, whereas performance in the F-DA-F condition (61%) was significantly lower from both, F(1,42) = 8.30; Mse = 250.8; p<0.01, and F(1,42) = 6.52; Mse = 250.8; p<0.05, respectively.

The above results replicate those obtained in previous studies showing that DA at encoding substantially reduces memory performance relative to full attention conditions, while DA at retrieval affects performance to a lesser degree, or as in the current experiment, not at all. However, memory performance in the second memory test for items from the first retrieval/test phase was significantly reduced in the DA at retrieval condition but not in the other attention conditions.

#### ACRT task performance

The ACRT task was performed both alone (under full attention), as baseline measure, and in the divided attention conditions in the DFF and FDF attention conditions. For the DFF group, the mean average RT was 740 msec (158) for the baseline condition and 899 (163), for the DA at encoding condition. For the FDF group, the mean RT was 821 msc. (121), for the baseline condition and 1580 (340) for the DA at retrieval condition.

A two-way ANOVA analysis revealed a significant effect for group [F(1,28) = 33.98, MSe = 64221, p<0.01], a significant effect for attention (Baseline vs. DA), [F(1,28) = 116.15, MSe = 27198, p<0.01], and a significant affect of the interaction [F(1,28) = 49.48, MSe = 27198, p<0.01]. Post hoc comparisons showed no differences between the baseline performance in the two groups [F(1,28) = 2.53, MSe = 19850, p<0.01], but a significant difference between the two groups in the DA condition [F(1,28) = 48.59, MSe = 71568, p<0.01]. Further follow-up comparisons showed a significant attention cost relative to the baseline condition in each of the two groups; DA at encoding was significantly slower than its baseline [F(1,28) = 7.00, MSe = 27198, p<0.05], as was DA at retrieval, [F(1,28) = 158.63, MSe = 27198, p<0.01]. The above results replicate previous findings, showing that encoding and retrieval are attention-demanding processes and that retrieval is more demanding than encoding.

#### Retrieval latency

Mean latencies across participants in each condition and test appear in Table 5. A two-way ANOVA showed a significant effect of attention/group [F(2,42) = 39.25, Mse = 73945, p<0.01], and test [F(1,42) = 115.59, Mse = 39102, p<0.01] as well as a significant interaction [F(2,42) = 46.42, Mse = 39102, p<0.01].

Follow-up interaction comparisons showed that in the first test, response latency in the DFF condition was not different than the one in the F-F-F condition [F<1], but that response latency in the FDF condition was significantly higher than in the F-F-F condition, [F(1,42) = 83.25, Mse = 89012, p<0.01]. However, in the second test, there were no differences in response latency between the two DA conditions and the F-F-F one [F(1,42) = 1.98, Mse = 24035, ns., for DFF vs. F-F-F], and [F(1,42) = 1.88, Mse = 24035, ns., FDF vs. F-F-F].

The retrieval latency in the first test replicates previous findings showing that divided attention at retrieval causes a substantial increase in retrieval latency relative to a full attention condition [30], [31].

Overall, the results of this experiment converge on those reported in the first three experiments in showing that DA at retrieval does affect memory performance, but that these effects can be measured only in a later memory test. This is the case even under incidental learning instructions where participants do not expect any of the tests.

## General Discussion

### Empirical Results

The results of the four experiments not only corroborate previous results regarding the effects of DA on learning/encoding and test/retrieval, but also provide novel insight into the effects of DA at retrieval on later memory performance (memory *for* rather than memory *at*). To summarize our major findings, the current experiments replicated previous results [5], [18], [7], [16], [8], showing that divided attention at encoding significantly downgrades memory performance, whereas divided attention at retrieval has a much smaller effect, if any, on memory performance. In the current study, this was demonstrated with different secondary tasks, an auditory CRT task (Experiments 1, 2, & 4), as well as a visual task (Experiment 3). The above-mentioned memory performance patterns were shown to be associated with greater attentional cost at retrieval than at encoding, as reported in Experiments 1, 2, and 4, when an auditory secondary task was used with a primary visual task.

Experiment 3, which used a visual secondary task, did not show larger attentional costs at retrieval versus encoding, which is similar to the results reported by Craik et al., [5]. Apparently, the auditory secondary task was more demanding (see baseline performance in Table 6), making it more difficult for participants to perform in conjunction with the retrieval task.

**Table 6:** Secondary task costs in msc. (after subtracting baseline condition performance) for encoding and retrieval, for all and across the experiments.

	DA at encoding	DA at retrieval
**Exp. 1**	458 (202)	759 (373)
**Exp. 2**	196 (93)	647 (248)
**Exp. 3**	26 (79)	96 (113)
**Exp. 4**	159 (89)	758 (317)
**Overall attentional cost**	**209**	**565**

The current study also provides further insight into response latency under DA conditions in a recognition paradigm. The results indicate that response latency at retrieval increases under DA, as found by Naveh-Benjamin and Guez [30], using a cued recall paradigm, and by Carrier and Pashler [32], who used a PRP paradigm. Yet, in contrast to results reported in Naveh-Benjamin & Guez [30] we found no evidence for delayed response latency when study was done under DA, and also in the second test, when attention was divided during the first test (see Table 7).

**Table 7:** Response latency in msc. at the two tests for the different targets and group/attention conditions.

		Full attention	DA at encoding	DA at retrieval
**Exp. 2**	**First test**	1201 (90)	1301 (161)	1756 (288)
	**Second test**	1148 (131)	1166 (116)	1187 (126)
**Exp. 3**	**First test**	1463 (308)	1377 (243)	1799 (152)
	**Second test**	1510 (213)	1417 (266)	1511 (353)
**Exp. 4**	**First test**	1348 (148)	1416 (147)	2342 (472)
	**Second test**	1255 (129)	1175 (162)	1332 (169)
**Overall latency at first test**		**1337**	**1364**	**1965**
**Overall latency at second test**		**1304**	**1252**	**1343**

* No latency measure was collected in Experiment 1.

Thus, the results suggest that in a recognition memory paradigm, latency delays may be present only when the secondary task and the memory responses are performed simultaneously, potentially creating a bottleneck, as suggested by Pashler and Johnston [31].

Most importantly, in addition to the traditional design with a study and a test phase, the current experiments added a second test phase that allows the assessment of the effects of DA at retrieval on later memory performance. Operating under the assumption that retrospective memory, by definition, refers to past experience, we suggest that in order to thoroughly assess the effects of DA at retrieval, we need to assess its effects on later memory performance. The results of all four experiments are very consistent in showing that DA during retrieval causes a clear decrease in memory performance for the information presented during retrieval (see Table 8).

**Table 8:** Percentage increase or decrease in memory performance relative to the F-F-F (full attention) condition in the first test (Studied items), and the second test (Tested items), in the different groups/attention conditions, for all the experiments.

		DA at encoding	DA at retrieval
**Exp. 1**	**Studied items** *	−37.9% (43.8)	+14.1% (46.7)
	**Tested items**	+9.4% (46.1)	−28.0% (32.4)
**Exp. 2**	**Studied items**	−33.9% (25.6)	−14.8% (30.6)
	**Tested items**	−1.3% (23.7)	−14.8% (27.5)
**Exp. 3**	**Studied items** *	−17.4% (37.1)	+14.8% (39.3)
	**Tested items**	+0.3% (23.6)	−22.3% (53.9)
**Exp. 4**	**Studied items**	−50.3% (38.9)	−6.6% (48.9)
	**Test items**	−0.8% (21.2)	−18.3 (32.2)
**Overall memory for** **studied items**		−**34.8%**	**+1.87%**
**Overall memory for** **tested items**		**+1.9%**	−**20.8%**

Full attention = 100%.

* In these experiments, we tested part of the learning of the studied items in the first test and other part in the second test. Here we average both.

The results reported in this paper extend those reported by Dudukovic et al., [22] in terms of the instruction manipulation used to enable us to minimize learning during test, the stimuli employed (verbal rather than pictorial), the mode of presentation (auditory and visual, rather than only visual), and the direct assessment of the interaction between attention and stimuli/test conditions. Our interpretation of the results is different than that of Dudukovic et al.; in their interpretation, they adhered to the notion of encoding as retrieval and retrieval as encoding, while we suggest a unique component (Episodic Register), independent of any specifically employed processes, which is disrupted by DA.

### Theoretical Issues

Previous research on encoding and retrieval processes using a divided attention manipulation pointed to some fundamental differences between the two (e.g., [33], ). Specifically, these studies concluded that encoding processes are controlled by and thus vulnerable to the effects of DA, whereas retrieval processes are more obligatory and ballistic, with divided attention interrupting them to a much smaller degree. The current results challenge these conclusions.

As suggested in the introduction, there seems to be a potential confound in previous studies between the time when memory is being tested (afterward vs. on-line) and the memory processes (encoding vs. retrieval). To eliminate this confound, we compared the effects of DA at encoding and retrieval using the same memory requirements “for” the information. This was done by testing the effects of DA at encoding and retrieval *after both* had occurred. The method employed used an initial test where the effects of DA during the original encoding could be assessed, as was done in previous studies, and then introducing a second test, where the effects of DA during retrieval in the first test could be assessed. Testing the above required the overcoming of several potential methodological pitfalls. These included 1) the possible reliance of participants in the second test on information from the study phase rather than from the first test, and 2) the fact that retrieving targets from the first test could enhance participants’ retention in comparison to the initial encoding phase.

To overcome these potential problems, we used a second memory test where participants had to retrieve the distractor items from the first retrieval phase. In those experiments (1 and 3)we used the original information from the study phase in the second test, ensuring that these items were not tested during the first test. We also made sure that memory for the distractors from the first test and assessed in the second test were not due to any effortful encoding of these distractors. We did so by providing only a brief exposure time for these distractors in the first test (Experiments 2 and 4), by ensuring that participants did not expect the second test (Experiment 3), and by using a pure incidental learning paradigm, where participants did not expect any memory test (Experiment 4).

All four experiments show that although DA at retrieval results in only small decrements, if at all, in on-line memory performance, such DA effects at retrieval have strong detrimental consequences in terms of the ability to retrieve this information at a later time. Such results can be interpreted to mean that both encoding and retrieval are interrupted by DA and that the asymmetry reported in the literature is an artifact of the test employed (“for” vs. “at”) to assess the effects at encoding and at retrieval.

However, even with these clear results there remains an open question as to what the specific component is, independent from the process involved (i.e. encoding/retrieval), that causes future memory decrease. The fact that there is a consistently large decline in later memory for information presented previously under DA at retrieval, even in cases where no strategic elaboration was used during the retrieval of this information, may suggest that what is affected by DA at retrieval is not the level of elaboration or any other strategic processes, but rather a degradation of the episodic buffer/episodic register capacity [19] that accounts for the consolidation of an event (whenever it involves encoding or retrieval) to episodic memory trace. This binding is also similarly disrupted under DA at encoding. In this sense, DA in general may affect the binding of components of an episode into the consolidated memory trace that in turn determines episodic recollection in the future. However, such an interruption by DA is not tied to encoding or retrieval *per se* but can be evoked at both stages.

Support for the notion that the interruption of DA at encoding is related to a disruption in episodic binding or consolidation is provided by studies that show that DA at encoding does not seem to decrease the level of elaboration [15], [16], self generation [15], or associative processing [27]. Additional support for the idea that DA affects the episodic consequence of an experience comes from research showing null effect of DA on semantic memory or on familiarity base processes [35]. Such an interruption of consolidation in the Episodic Register may be related to frontal – MTLH connections, in line with Moscovitch [36] who suggested that DA causes a connection failure between the frontal areas and the MTLH, causing episodic awareness failure or a failure in creating a binding index for future episodic remembering. Lately, imaging study focusing specifically on EB found that episodic buffer seems to engage frontal networks and the hippocampus [37].

## Summary

In this study, we evaluated the conceptualization of encoding and retrieval processes established in studies that used a divided attention (DA) paradigm. The current work suggests that the inherent asymmetry between encoding and retrieval based on the DA literature, may be understood as a confounding within the DA paradigm used. Instead, we present a more symmetric pattern between encoding and retrieval by targeting the above confound. Our results consistently show that DA at retrieval strongly disrupts later memory for the retrieved episode, similar to the effects of DA at encoding. We suggest that a conceptualization in terms of an episodic-buffer or an episodic-register can help in our understanding of the missing element of the effect of DA on memory.
